# Supplementary figures and images for: Amphibalanus amphitrite begins exoskeleton mineralization within 48 hours of metamorphosis
Source: R Soc Open Sci. 2020 Sep 30;7(9):200725. doi: 10.1098/rsos.200725 (PMC7540746; doi:10.1098/rsos.200725)

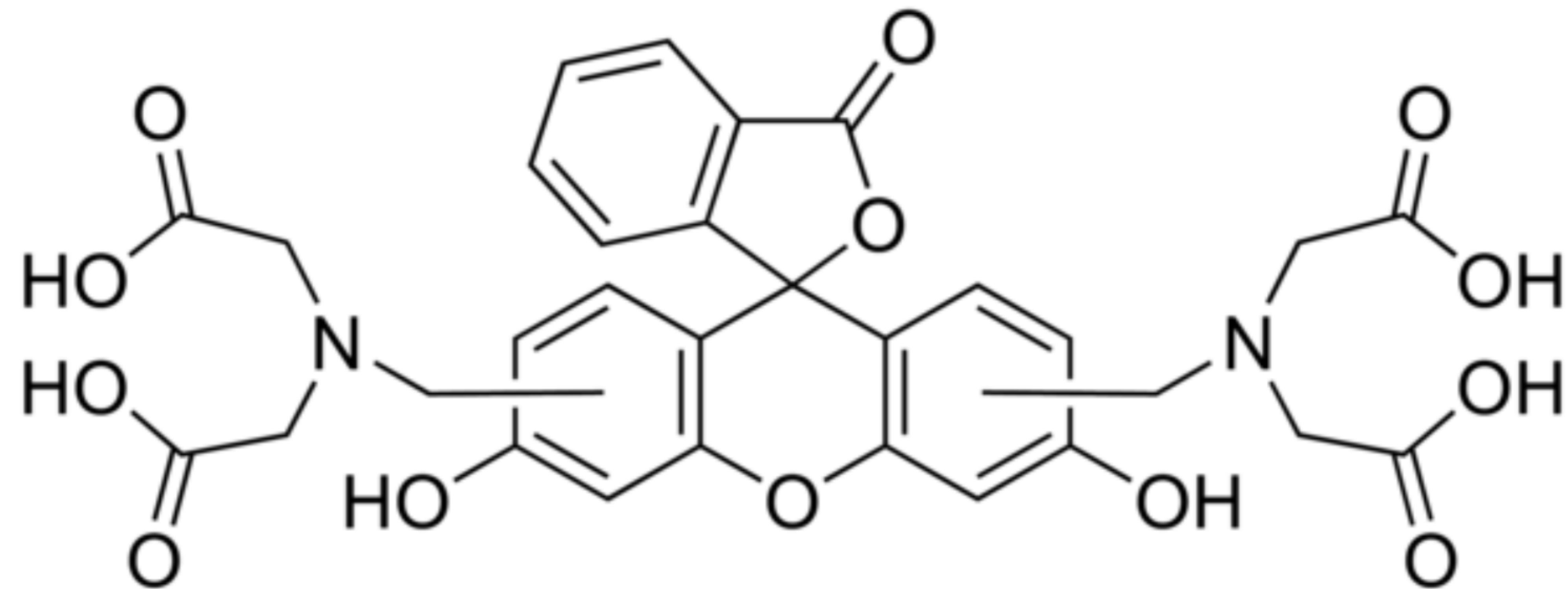

Supplement: Figure S1 [file rsos200725supp1.pdf]

*Balanus amphitrite* confocal intensity

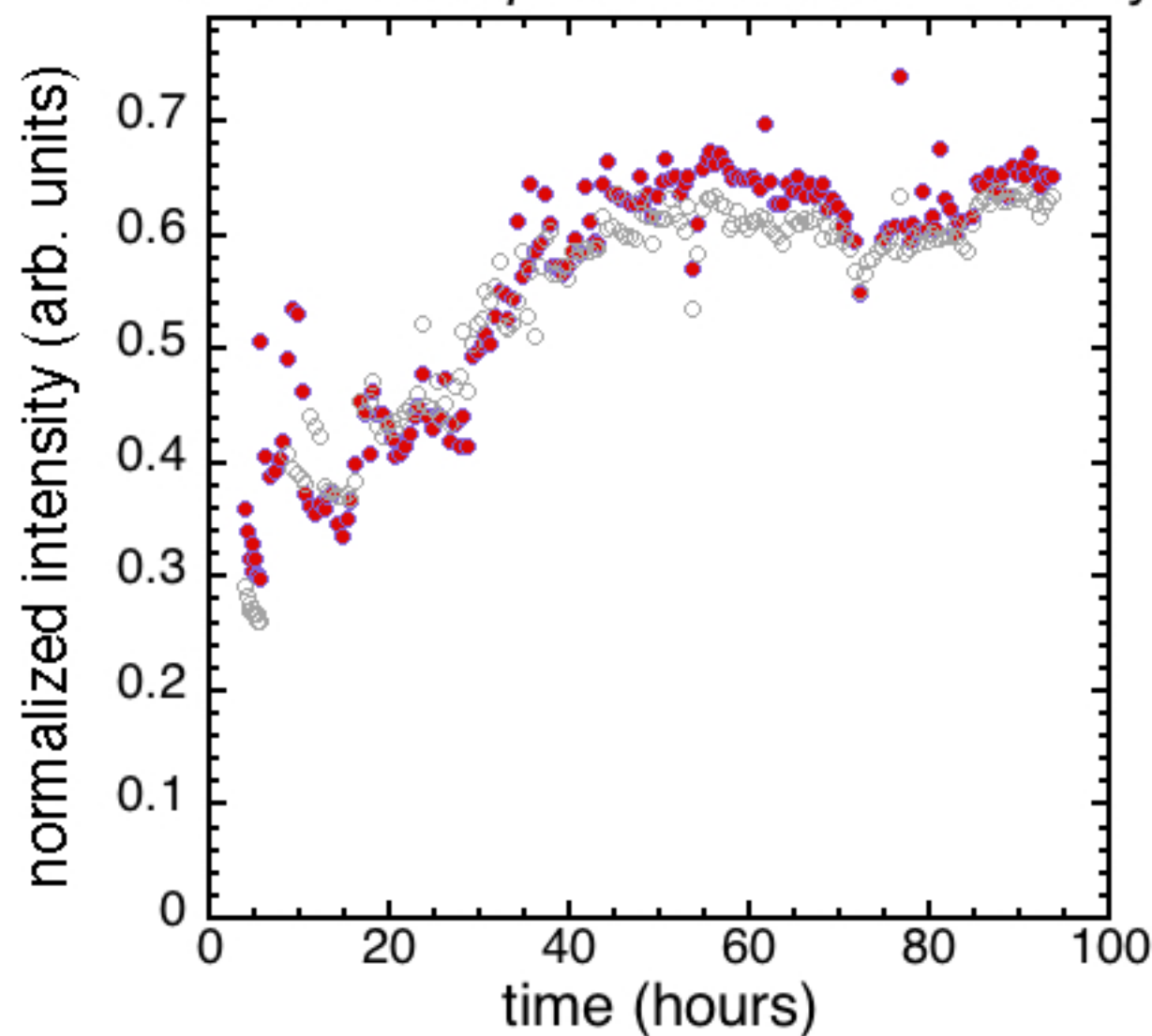

Supplement: Figure S2 [file rsos200725supp2.pdf]

*Balanus amphitrite* baseplate growth

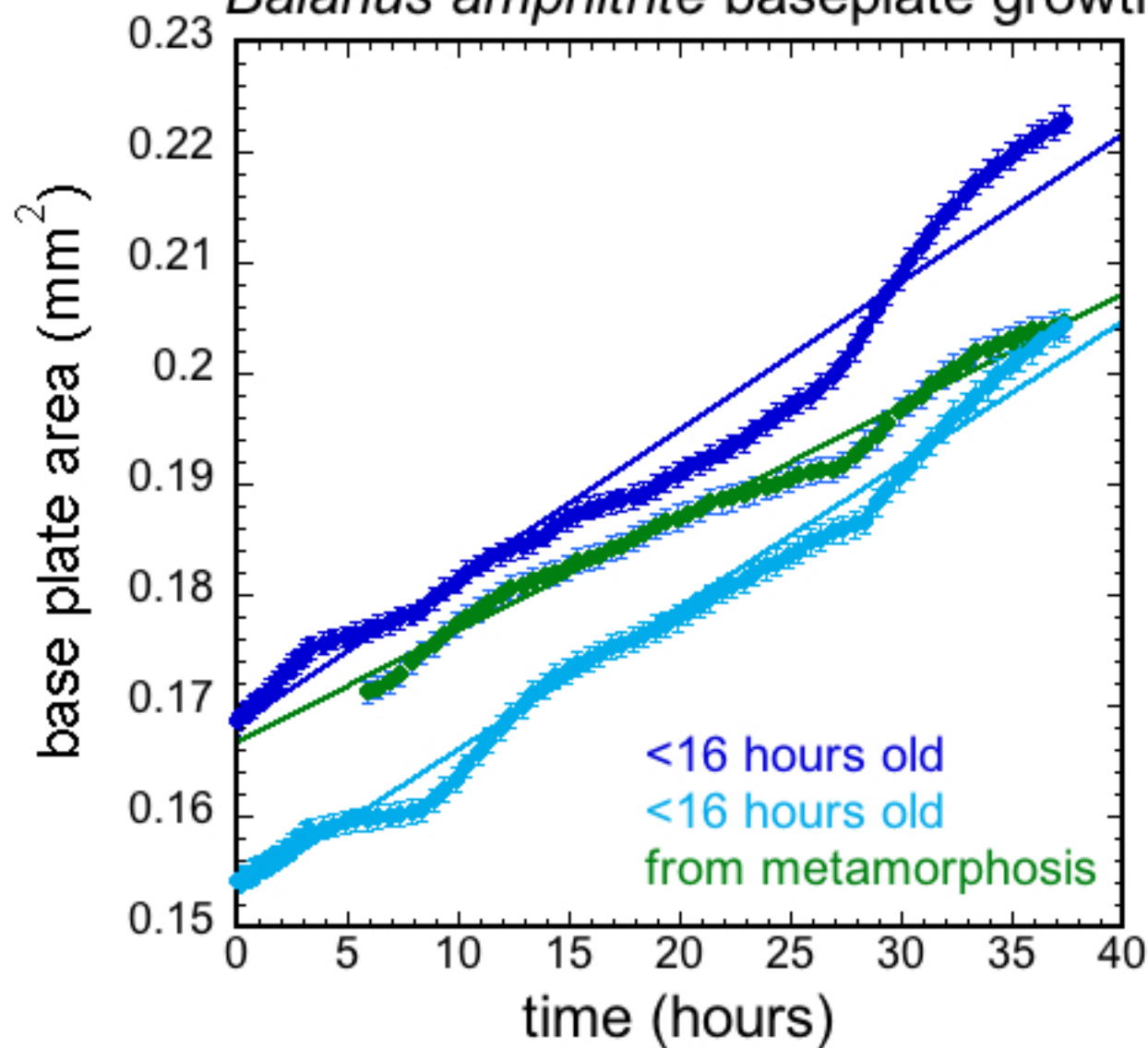

Supplement: Figure S3a [file rsos200725supp3.pdf]

*Balanus amphitrite* baseplate growth

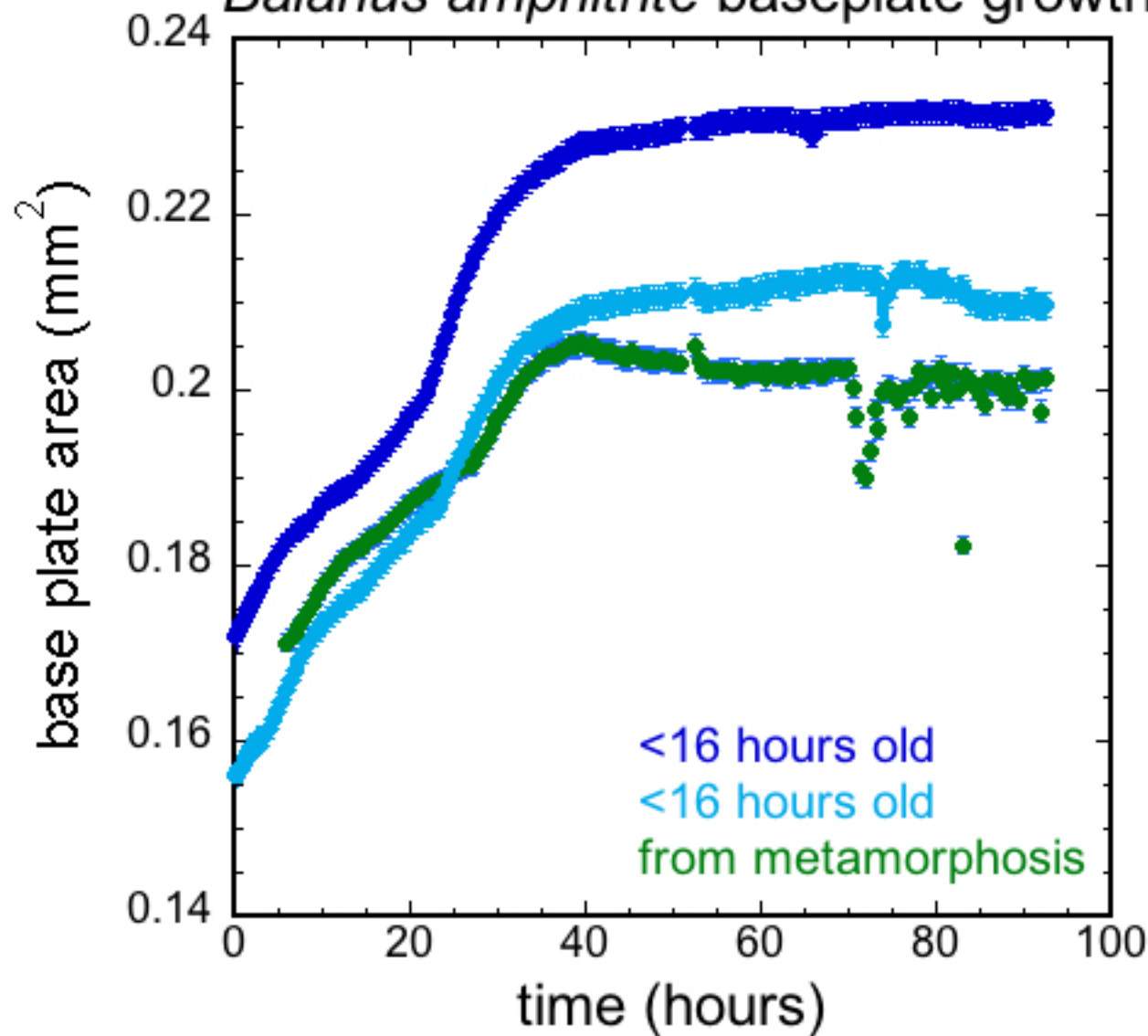

Supplement: Figure S3b [file rsos200725supp4.pdf]

*Balanus amphitrite* exoskeleton height

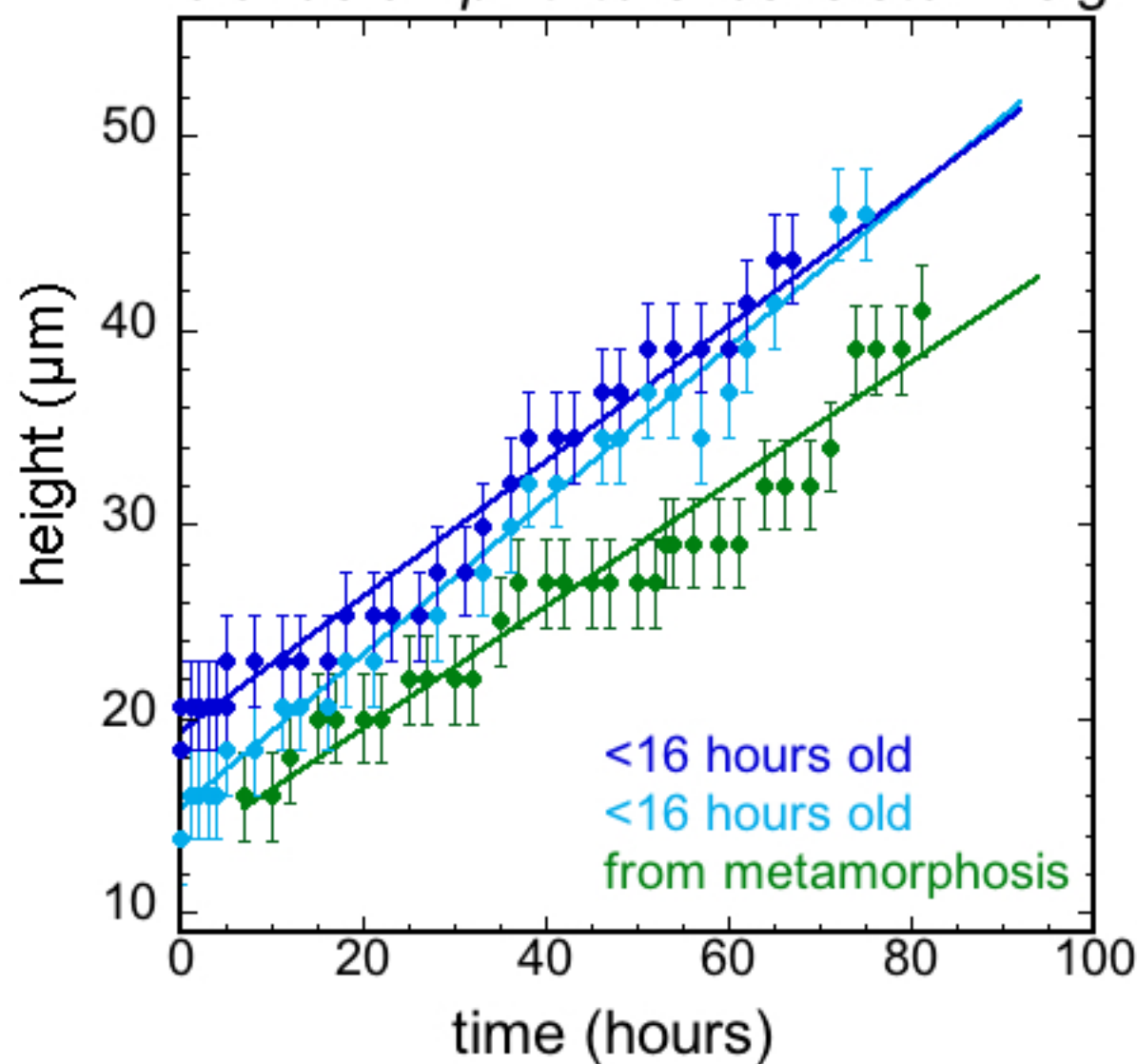

Supplement: Figure S4 [file rsos200725supp5.pdf]

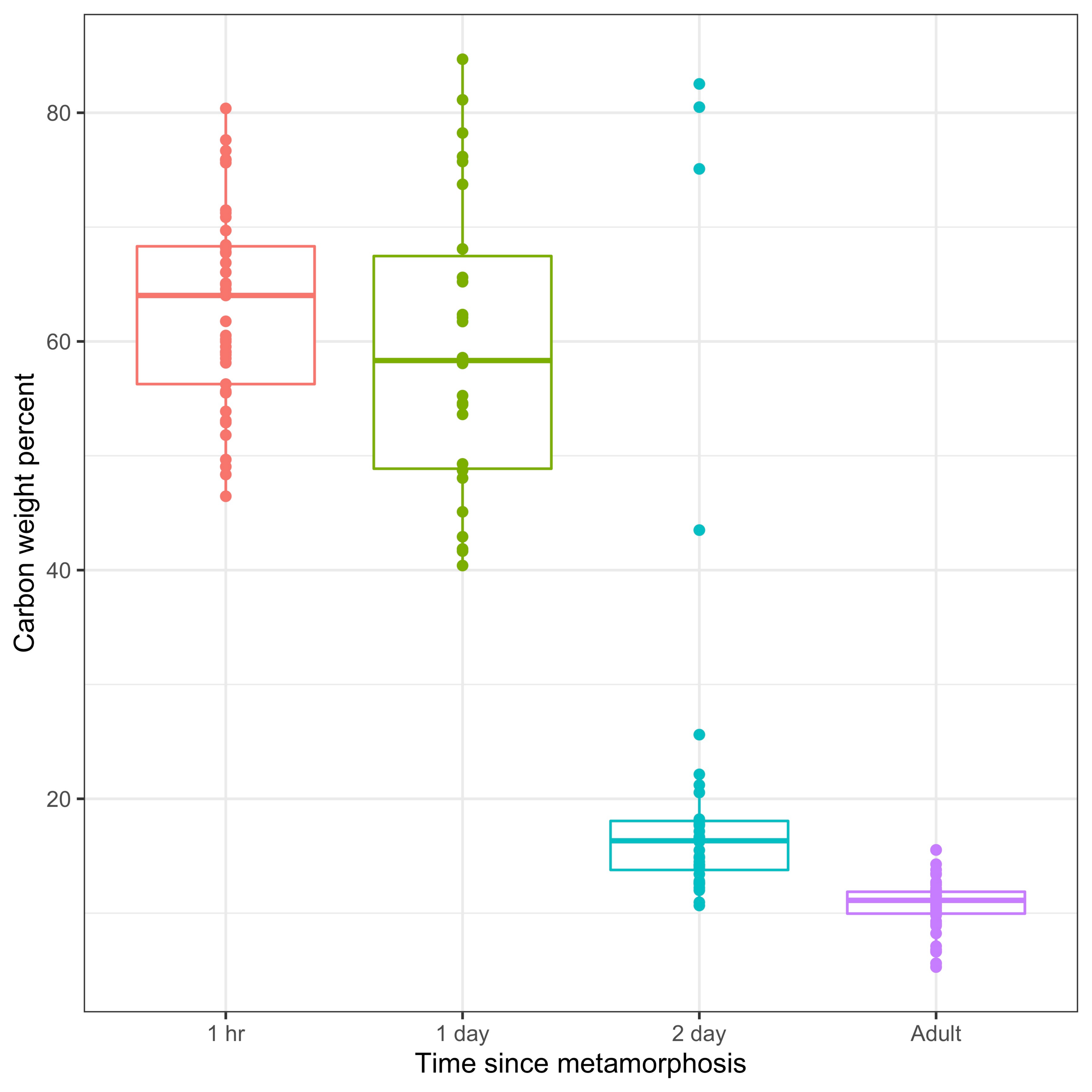

Supplement: Figure S5C [file rsos200725supp6.pdf]

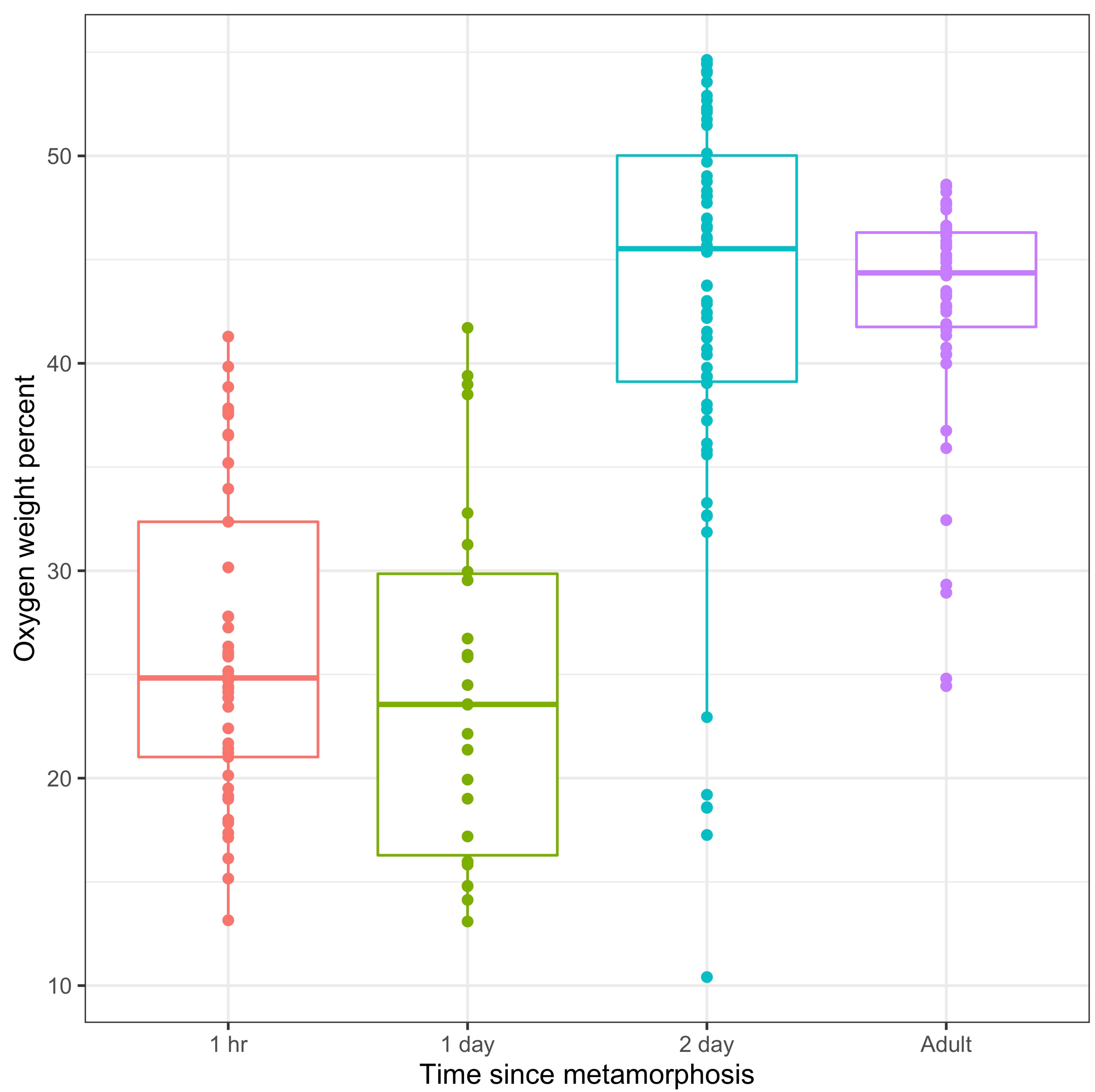

Supplement: Figure S5O [file rsos200725supp7.pdf]

Silicon background Raman Spectrum

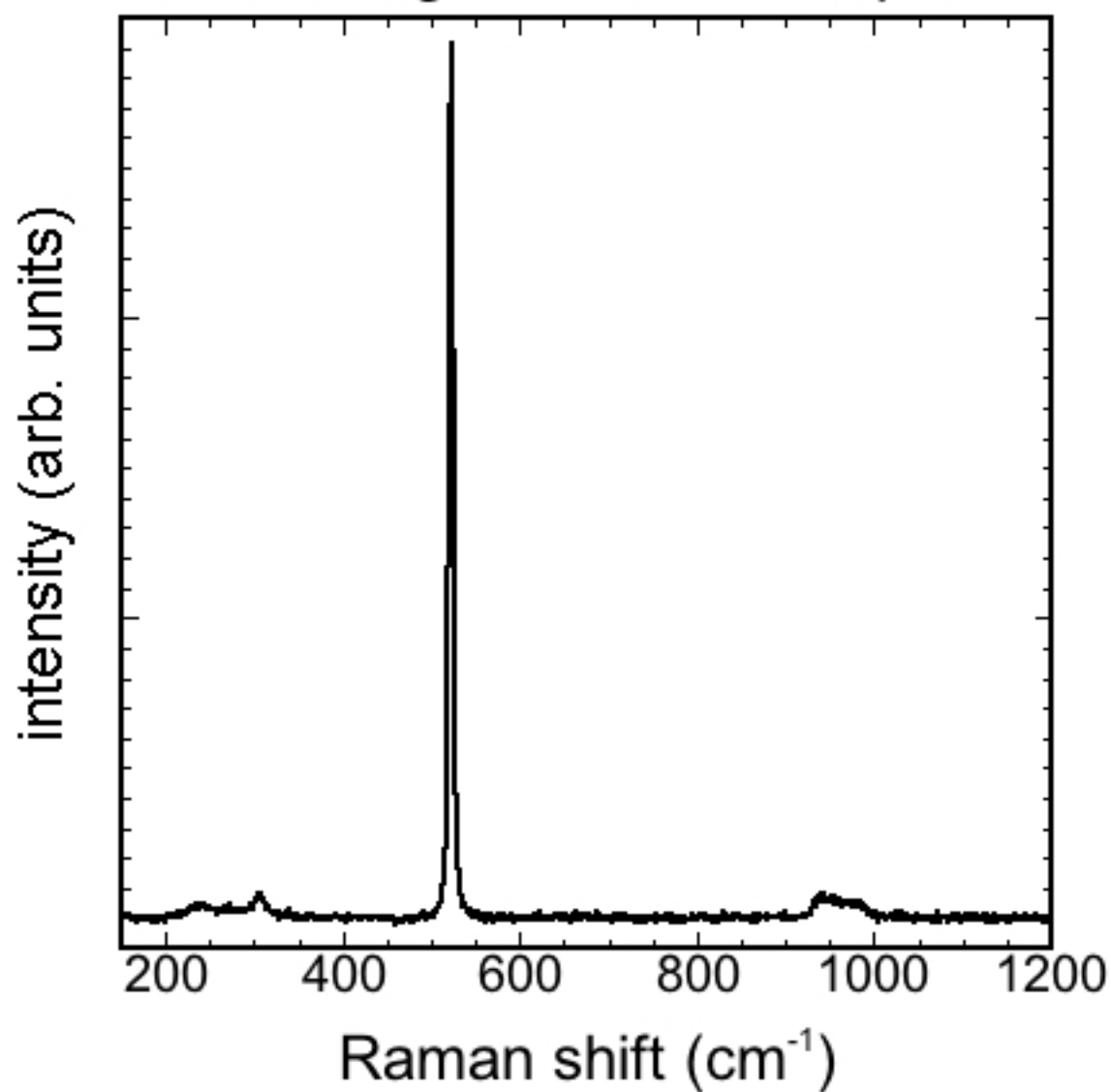

Supplement: Figure S6 [file rsos200725supp9.pdf]
